# Clinicopathological Correlations in Enucleated Globes of Late-Stage Coats Disease with a Review of the Literature

**DOI:** 10.1007/s44197-022-00068-y

**Published:** 2022-09-29

**Authors:** Hala A. Helmi, Abdullah Jammah, Azza MY. Maktabi, Saleh AlMesfer, Hind M. Alkatan

**Affiliations:** 1 Department of Ophthalmology, McGill University, Montreal, Canada; 2 Ophthalmology Department, King Faisal Specialist Hospital and Research Center, Riyadh, Saudi Arabia; 3 College of Medicine, King Saud University, Riyadh, Saudi Arabia; 4 Pathology and Laboratory Medicine Department, King Khaled Eye Specialist Hospital, Riyadh, Saudi Arabia; 5 Pediatric Ophthalmology Division, King Khaled Eye Specialist Hospital, Riyadh, Saudi Arabia; 6 Department of Ophthalmology, College of Medicine, King Saud University, Riyadh, Saudi Arabia; 7 Department of Pathology, College of Medicine, King Saud University, Riyadh, Saudi Arabia; 8 King Saud University Medical City, College of Medicine, King Saud University, Riyadh, Saudi Arabia

**Keywords:** Coats disease, Retinoblastoma, Leukocoria, Enucleation, Globe, Subretinal exudate

## Abstract

**Background:**

Coats disease may cause diagnostic dilemma because of its variable clinical presentation that can be suspicious of retinoblastoma. Late sequelae of the disease are blinding with possible enucleation. We demonstrate the main histopathological findings of Coats enucleated eyes with literature review.

**Methods:**

This was a retrospective study of all enucleated globes diagnosed as Coats disease over 30 years and were reviewed by two pathologists. The corresponding demographic data, clinical presentation, pre-operative clinical impression, and indication for enucleation were collected. Descriptive analysis of our own series data was performed. Our findings were then correlated to published data that were collected from 1983 to 2021 from the PUBMED database in English-written language. Shields classification was used as an inclusion criterion for the published reports to be analyzed.

**Results:**

We had seven enucleated globes with Coats disease. Mean age at presentation was 3.2 years (range 3 months to 9 years). Male predominance was observed in 6 and all cases were unilateral. Strabismus was the most common initial presentation (57%, *n* = 4), followed by leukocoria (43%, *n* = 3). Indication for enucleation was mostly suspected retinoblastoma (57%, *n* = 4). Four eyes were classified as stage 4, and 2 had advanced stage 5 changes. Histopathologically, subretinal fluid with lipid-laden macrophages was seen in all cases, the anterior chamber was shallow in 5/7 with angle neovascularization in 2/7. Telangiectatic vessels were clearly observed in 4/7.

**Conclusion:**

Coats disease is a potentially visually disabling disease that is mostly unilateral in 95%, has male predominance of 81%, and wide age range with a mean of 17 years. In Saudi Arabia, the disease seems to present at younger age, tends to be more advanced, and may be indistinguishable from retinoblastoma at the time of diagnosis. Shields staging of Coats is highly recommended to be followed clinically to unify the pathways for treatment and to correlate the concluded outcomes.

## Introduction

Coats disease is an idiopathic non-hereditary eye condition that was first described by Scottish ophthalmologist George Coats in 1908 as unilateral retinal telangiectasia and aneurysms with retinal exudation [1–3]. The disease is known to have male predominance, is more common at a young age, and has no known association with systemic conditions [1–3]. The pathogenesis of Coats disease is not fully understood, however, multiple studies have proposed that the telangiectatic blood vessels are more permeable, causing leakage of lipoproteins into the retina [4]. The accumulation of lipoproteins leads to a break in the external limiting membrane of the retina which in turn causes non-rhegmatogenous retinal detachment [4] As further accumulation of the lipo-proteinaceous material takes place, the non-rhegmatogenous retinal detachment progresses to become a bullous retinal detachment [4]. In this case series, we aim to present the histopathological findings of enucleated globes with the tissue diagnosis of Coats disease, investigate the indications for enucleation, and present the clinicopathological correlation irrespective of the age at the time of enucleation. We also reviewed the literature on Coats disease to demonstrate useful conclusions in relation to demographics, clinical diagnosis, and outcome of treatment.

## Materials and Methods

All enucleated globes that were received at the Pathology & Laboratory Medicine Department at a tertiary eye hospital and diagnosed as Coats disease over a period of the last 30 years were reviewed by 2 pathologists. The corresponding demographic data, clinical presentation, pre-operative clinical impression, and indication for enucleation were collected to identify cases that underwent enucleation with the assumption of retinoblastoma, assess the severity of clinical presentation, and perform clinicopathological analysis. Descriptive analysis of our data was carried on throughout the series. The study was conducted in accordance with relevant guidelines/regulations and in accordance with the Declaration of Helsinki. The research project was granted an expedited approval as a retrospective type of study by the Human Ethics Committee/Institutional Review Board (HEC/IRB) at King Khaled Eye Specialist Hospital on 10th May 2022 with an approved Collaborative agreement with King Abdulaziz University Hospital (RP-22052-R). A written general informed consent was obtained from all participants and/or their legal guardians for anonymous use of their data. This research does not involve drug trials nor human transplantation.

### Literature Review

Our findings were then correlated to published data on this rare disease to compare our demographics and disease severity to extracted summarized data from previously published series in the English-written literature. We performed a literature review on published studies of Coats disease from 1983 to 2021 from the PUBMED database in English, using the keywords “Coat's disease” and “Coats”, which yielded seven studies: [3, 6–10]. Excluded studies were those that only included a specific age group. One study was excluded to avoid a skewed deviation in our review as it only included adult-onset Coats disease: [5]. The six studies included a total of 964 patients (1009 eyes) presenting with unilateral or bilateral involvement and are summarized in Table 1 [3, 6–10]. Staging of all cases as applicable from 5 studies is summarized in Table 2.

**Table 1:** The number of patients and eyes in six major studies

	Number of patients	Number of eyes
Shields et al. [3]	150	158
Tarkkanen et al. [6]	24	25
Al-Qahtani et al. [7]	92	97
Kang et al. [8]	67	71
Rishi et al. [9]	280	307
Shields et al. [10]	351	351
Total	964	1009

**Table 2:** Shields classification at presentation of patients with Coats disease from five studies [6–10]

Shields classification at presentation [10]	Stage 1	11 (1.3%)
	Stage 2	280 (33.9%)
	Stage 3	460 (55.7%)
	Stage 4	57 (6.9%)
	Stage 5	7 (0.9%)
	Unknown	11 (1.3%)

The study by Shields et al. was excluded from the above analysis because they did not include the Shields classification in their study [3]

## Results

We present seven patients with unilateral Coat’s disease, confirmed by histopathology following enucleation ranging in age at presentation from 3 months to 9 years with a mean age of 3.2 years. Among our seven enucleated cases of Coats disease, six of them were male. All cases were unilateral with no systemic associations. The left eye was affected in four cases, and the right eye in three cases. Strabismus was the most common initial presentation to our tertiary eye center (57%, *n* = 4), followed by leukocoria (43%, *n* = 3). The demographic details and clinical features are summarized in Table 3.

**Table 3:** Summarized demographics and clinical features of patients with Coats disease from the same six studies with comparison to our series

	Literature review cases		Our cases (enucleated)	
Mean age (range)	16.895 years (0.08–80 years)		3.2 years (0.25–9 years)	
Gender	Male	784 (81%)	Male	6 (86%)
	Female	180 (19%)	Female	1 (14%)
Presentation	Unilateral	916 (95%)	Unilateral	7 (100%)
	Bilateral	48 (5%)	Bilateral	0 (0%)
Presenting sign	Decreased VA	565 (59%)	blindness	2 (29%)
	Strabismus	253 (26%)	Strabismus	4 (57%)
	Leukocoria	112 (12%)	Leukocoria	3 (43%)
	Asymptomatic	29 (3%)	Pain	2 (29%)

*VA* visual acuity

Indication for enucleation was suspected retinoblastoma (57%, *n* = 4), blind painful eye (29%, *n* = 2), and one eye was enucleated due to bullous retinal detachment (RD) with poor vision. The indications for enucleation and histopathological findings of our cases are summarized in Table 4.

**Table 4:** Pre-operative diagnosis, indication for enucleation and histopathological findings of our enucleated cases

Case # Eye	Pre-operative diagnosis	Age at enucleation	Indication for enucleation	Histopathological posterior segment findings
Case 1 OS	Coats disease with exudative retinal detachment	14 months	High bullous retinal detachment	Organizing subretinal exudation with lipid filled spaces and angular cholesterol clefts, some of which show giant cell formation
Case 2^a^ OD	Blind painful eye status post multiple surgeries for congenital glaucoma	7 years	Blind painful eye	Total retinal detachment with abnormal enlarged vessels. Subretinal proteinaceous fluid with cholesterol clefts, giant cells, and scattered macrophages. Choroidal detachment
Case 3 OS	Coats disease with panuveitis and exudative retinal detachment	9 years	Blind painful eye	Subretinal proteinaceous transudate, lipid vacuoles, epithelioid cells, massive subretinal and epiretinal fibrous proliferation, and abnormal retinal blood vessels with thickened walls. Uveal infiltration by lymphocytes
Case 4 OD	Retinoblastoma with total funnel-shaped retinal detachment	3 months	Inability to rule out retinoblastoma clinically	Total retinal funnel-shaped detachment, thickened, peripheral retina telangiectatic blood vessels, and subretinal exudation, lipid, and hemosiderin laden-macrophages
Case 5 OS	Retinoblastoma vs. Coats disease with total funnel-shaped retinal detachment	14 months	Inability to rule out retinoblastoma clinically	Total funnel-shaped retinal with empty spaces of dissolved lipid. Subretinal proteinaceous substance and lipid-laden macrophages
Case 6 OD	Coats disease vs. Retinoblastoma vs. Medulloepithelioma	8 months	Inability to rule out retinoblastoma clinically	Total funnel-shaped retinal detachment, Posterior cavity is filled with proteinaceous substance, aggregates of lipid-laden macrophages, and few cholesterol clefts
Case 7 OS	Retinoblastoma vs. Coats Disease with shallow retinal detachment	3 years	Inability to rule out retinoblastoma clinically	Atrophy with the focal gliosis, abnormal vascular pattern, and entrapped proteinaceous fluid. Subretinal cholesterol clefts, giant cells, and foamy histiocytes

*OS* left eye; *OD* right eye

^a^Had also total funnel retinal detachment on B-scan

## Discussion

Coats disease is a rare condition that usually affects males at a young age, most likely in the first two decades of life. [3, 6–10]. In our review, we similarly found a male predominance which accounted for 81% of cases (*n* = 784). The mean age at presentation among all studies was 17 years (age range was 0.08–80 years) [3, 6–10]. The disease may affect adults, but usually with a less advanced stage in comparison to those affected at a younger age [11, 12]. Our enucleated cases with Coats presented in the pediatric age group with a mean age of 3.2 years and they were considered to be advanced based on the presentation and histopathological findings. The yearly incidence of Coats disease was estimated to be 0.09:1,000,000 [13].

### Clinical Features

Coats disease is more commonly unilateral, as was correlated in our review, where the percentage of unilateral cases was 95% (*n* = 916) [3, 6–10]. It can have a wide range of clinical presentation. In our review, we found that the most common presenting sign was decrease in visual acuity (59%, *n* = 565), followed by strabismus (26%, *n* = 253), and leukocoria (12%, *n* = 112). A minority of patients (3%, *n* = 29) were diagnosed on routine examination as they were asymptomatic [3, 6–10]. Our study contrasted the literature in that decreased vision was not as well documented, possibly owing to the younger age at presentation. Thus, strabismus was the most common initial presentation in our study (57%, *n* = 4), followed by leukocoria (43%, *n* = 3). We had two advanced cases (cases 2 and 3) presenting with severe visual loss and complicated clinical presentation; a 7-year-old male presenting with pain and tearing in his right blind eye. He had presumed congenital glaucoma for which he underwent multiple surgeries and was found to have a total funnel-shaped retinal detachment on examination. The other was a 9-year-old male who presented to our emergency with left eye pain following blunt trauma to the same left blind eye. This patient was already known to have pan-uveitis and exudative retinal detachment and was following in another hospital but was not accurately diagnosed as a case of Coats disease. Based on Shields classification summarized in Table 2, the stages at presentations in their large clinical series, were stage 1 (1.3%, *n* = 11), characterized by telangiectasia only, stage 2 (33.9%, *n* = 280), characterized by telangiectasia and exudation, stage 3, which was the most common (55.7%, *n* = 460), characterized by retinal detachment without glaucoma, stage 4 (6.9%, *n* = 57), characterized by total retinal detachment with glaucoma, stage 5 (0.9%, *n* = 11), characterized by advanced end-stage changes and unknown (1.3%, *n* = 11) [4, 6–10]. In another study of 158 eyes by Shields et al. [3] the previous Shields classification was not used, however, their analysis of the posterior segment findings concluded that 100% (*n* = 158) of eyes had retinal telangiectasia which is known to be a common sign in Coats disease [3]. Other common findings from their study included intraretinal exudation (99%, *n* = 157) and partial or total exudative RD (81%, *n* = 128) [3]. Most of our enucleated globes represented late advanced stage of the disease (71%, *n* = 5). Four of these had total funnel-shaped retinal detachment (stage 4), and two had advanced end-stage changes (stage 5) confirmed by ultrasound and histopathological examinations (owing to difficulty in clinical assessment because of media opacity). Only one patient had the typical fundus finding of telangiectatic vessels and retinal exudation clinically, which was classified as stage 3.

### Diagnosis

Establishing the diagnosis of Coats can be a difficult task. Studies showed that as many as 50% of the cases of Coats were misdiagnosed at first. The disease was most commonly misdiagnosed as retinoblastoma [14]. Other differential diagnoses include Norrie disease, familial exudative vitreoretinopathy, retinopathy of prematurity, persistent hyperplastic primary vitreous, retinal capillary hemangioma, retinal cavernous hemangioma, incontinentia pigmenti, retinitis pigmentosa and toxocariasis [15]. The diagnosis of Coats disease is made by clinical presentation, fundoscopic examination, ultrasound, fluorescein angiography, computed tomography, and optical coherence tomography [10, 16, 17]. The clinical hallmarks of Coats disease include exudative RD, irregularly dilated telangiectatic vessels and peripheral nonperfusion [17, 18]. The main indications for enucleation of an affected eye with Coats disease are painful eye due to secondary glaucoma and when the diagnosis of retinoblastoma cannot be ruled out [1]. In our series, globes were enucleated mostly because of risk of retinoblastoma in 57% of cases (*n* = 4), followed by blind painful eye (29%, *n* = 2); one of which had a total funnel-shaped retinal detachment while the other had an exudative RD and pre-phthisical eye. Lastly, 1 case had enucleation due to a high bullous RD in an eye with poor vision but no pain as per Table 4.

### Histopathology

**Fig. 1:**
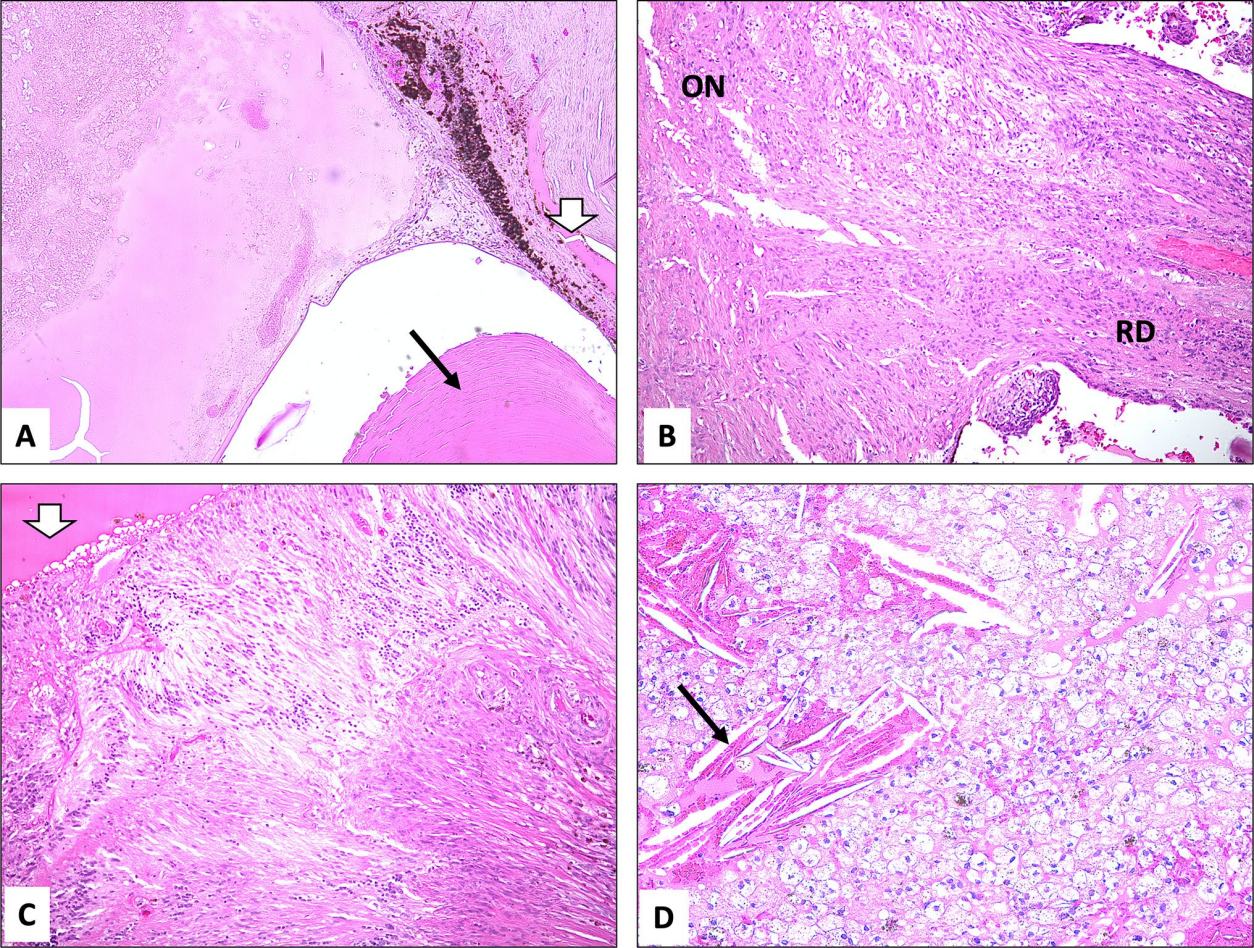
**A** Obliterated anterior chamber angle (white arrowhead) with atrophic iris and retro-lental (black arrow pointing at the lens) proteinaceous hemorrhagic fluid (original magnification X50 Hematoxylin & eosin). **B** An atrophic optic nerve head (ON) with overlying total retinal detachment (RD) (original magnification × 100 Hematoxylin & eosin). **C** Retinal shallow detachment with subretinal fluid (white arrowhead) and abnormal retinal blood vessels (original magnification × 100 Hematoxylin & eosin). **D** The subretinal fluid with fresh hemorrhage and cholesterol clefts (black arrow) indicating old hemorrhage (original magnification × 200 Hematoxylin & eosin)

**Fig. 2:**
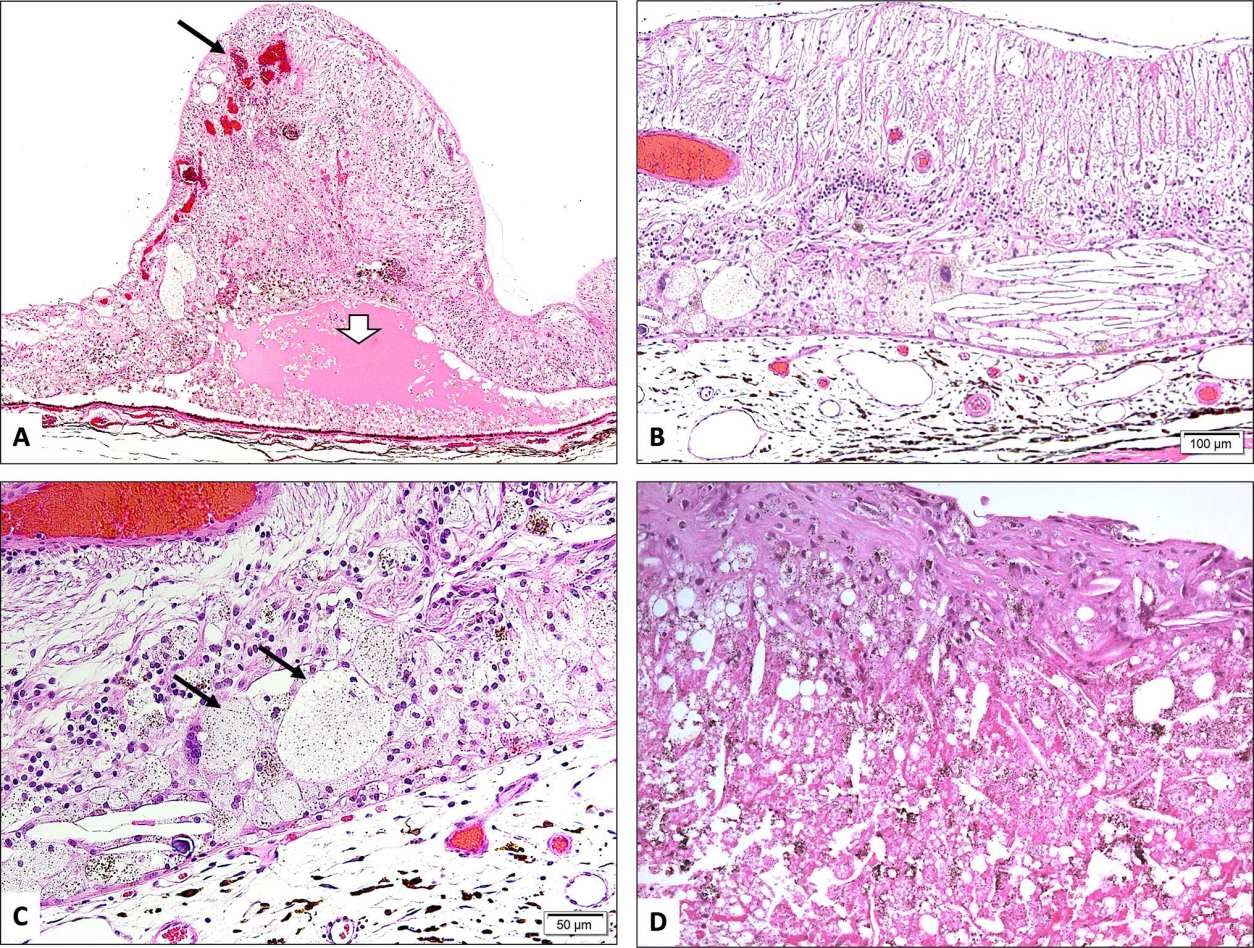
**A** A focus of shallow retinal detachment with subretinal fluid (white arrowhead), abnormal retinal vasculature and telangiectatic blood vessels (black arrow) near the ora serrata (original magnification × 100 Hematoxylin & eosin). **B** Cholesterol clefts and foamy histiocytes within the sensory retina (original magnification X100 Hematoxylin & eosin). **C** Higher power of the foamy macrophages (black arrows) with giant cell formation (original magnification × 200 Hematoxylin & eosin). **D** Subretinal exudate with cholesterol clefts and pigment dispersion in another case of Coat’s Disease (original magnification × 200 Hematoxylin & eosin)

There is a lack of studies that discuss the histopathological features of Coats disease in detail, most likely as a result of the new conservative methods of treatment [19]. The few reports of enucleation done in Coats disease have provided us with some pathological knowledge. Coats disease globes mostly show variable retinal detachment that can be bullous reaching to the lens in some cases. In advanced cases of Coats, findings may include displacement of the lens–iris diaphragm, blockage of the anterior chamber angle, hemorrhage, and detached retina. The subretinal space is filled with thick yellow lipo-proteinaceous exudation, sometimes with clumps of yellow material [4]. In our series, the anterior chamber was obliterated and/or shallow in 5/7 globes, indicating the advanced stage of the disease (Fig. 1A). A fibro-neovascular membrane along the iris surface, with secondary closure of the anterior chamber angle, was evident in 2 enucleated eyes because of neovascular glaucoma and pan-uveitis (Cases 2 and 3). Posterior segment examination of the globes in our series has shown variable RD (mostly total in 4/7) with eosinophilic subretinal fluid (Fig. 1B,C). Lipid-laden macrophages were present in the hemorrhagic subretinal fluid in all cases with or without cholesterol clefts (Fig. 1D). The peripheral retinal blood vessels were described as abnormal with larger than normal caliber corresponding to the areas of telangiectasia that might be seen clinically in 4/7 cases (Fig. 2A). The sensory retina was thickened by homogeneous acellular material, typical of lipo-proteinaceous exudation with foamy cells in 6/7 globes (Fig. 2B,C), which had similar appearance to the subretinal exudate (Fig. 2D). The origin of the lipid-laden cells has been the subject of some controversy. It is possible that they are derived from the blood or the pigment epithelium. Ultrastructural studies have not been conclusive as to their origin [4]. Tarkkanen et al. presented in their manuscript 24 patients (25 eyes) with Coats disease, of whom nine blind eyes were enucleated due to total exudative RD or untreatable secondary glaucoma [6]. They described the histopathological findings of the retina in their enucleated eyes which included bullous total RD with deep and subretinal exudates, massive gliosis, retinal disorganization, fresh and/or old hemorrhages, ghost cells and cholesterol crystals [6]. The retinal vasculature showed dilation and hyalinization [6]. This is very similar to the findings in our series as shown in Table 4.

### Treatment and Outcome

Treatment options for Coats include laser photocoagulation, cryopathy, vitrectomy, and intravitreal anti-VEGF therapy [20]. The natural course of Coats disease is progressive, but early stages of the disease can be followed with close observation or treated with laser photocoagulation. Late complications include neovascular glaucoma, RD, anterior chamber cholesterolosis, vitreous hemorrhage, and retinal bleeding from abnormal blood vessels [3, 4]. The prognosis of the disease varies depending on the stage at which the patient presents, however, the overall visual prognosis is poor [4]. Patients who present with stage 1 usually have a favorable visual outcome. The prognosis of patients who present with retinal detachment in stage 2 or stage 3 disease depends on the extent of the detachment and the involvement of the fovea [4]. The actual outcome in our series was not possible to be studied since we have only included enucleated globes, which were expected to have advanced disease with poor prognosis. In a large clinical-based study on 97 eyes from 92 patients diagnosed as Coats disease in the same tertiary center in Saudi Arabia, the most common stage was found to be stage 3 in more than half of the eyes (56%), which was uniformly concluded in our analysis as per Table 2. This might indicate a relatively more aggressive phenotype of the disease among Coats disease cases in our population [7]. The treatment modalities included laser photocoagulation in 64% of eyes, followed by cryotherapy, intravitreal agents, and surgical drainage in the order of frequency. About one third of their eyes underwent combination treatment and poor outcome was correlated to lower age (less than 10 years), visual acuity of 20/200 or less, advanced stages of the disease (stages 3 and 4), and the presence of subretinal fluid including the fovea [7].

Despite treatment, Coats disease in general is progressive in its nature and the result of advanced stages is usually blindness of the affected eye [20]. In Saudi Arabia, the above-mentioned clinical series demonstrated an enucleation rate of 13% out of the 97 eyes with indications that closely resemble those found in our series [7]. The histopathological findings in our enucleated globes clearly demonstrated the late sequelae of the disease.

Limitations to our study include the small number of cases owing to the rarity of this condition, and the inclusion criteria used that might not be totally representative of the clinical spectrum of the disease in Saudi Arabis but rather the complicated cases that unfortunately ended up by enucleation. Despite these limitations, the tissue findings in our series clearly demonstrate the extent of changes that can occur as a natural process of this disease due to the development of neovascular glaucoma and inflammation. Also, our collected data that were summarized and analyzed in our review have yielded useful conclusions that correlate with the literature and highlight the need of early and accurate diagnosis to minimize the risk of avoidable enucleation, and misdiagnosis in the pediatric population.

## Conclusions

Coats disease is a rare, and visually disabling disease that is predominantly unilateral, presents at wide age range but favors the young, and has a strong male predilection of 81%. Shields staging of Coats is highly recommended to be followed clinically to unify the pathways for treatment and to correlate the anticipated outcomes. More than half the cases present at stage 3 of the disease followed by stage 2 in about one third of cases, mostly with decreased vision. Early identification and conservative treatments may allow globe salvage to preserve vision. Despite the currently advanced diagnostic modalities, instances of eyes being enucleated with a suspected diagnosis of retinoblastoma remains prevalent and concerning. In Saudi Arabia, we further evidence that the disease presents at a younger age, which tends to be more advanced at the time of diagnosis with relatively poor vision. The natural pathological history of such these early cases shows neovascular glaucoma, reduced visual function, and the peculiar histopathological changes observed within the ocular structures. More than half of the enucleations were performed due to leukocoria and suspected retinoblastoma, necessitating a higher level of awareness of general ophthalmologists to the wide range of clinical presentations of Coats disease. We hope that both a higher level of suspicion, as well as a stronger ability to narrow the diagnosis will result in earlier and improved diagnostic accuracy, improved early management, and ultimately higher rates of globe salvage.

## Data Availability

The data used in this study is available with the corresponding author and can be provided upon request.

## Abbreviations

RD: Retinal detachment
VA: Visual acuity
OD: Right eye
OS: Left eye
